# Facilitators and barriers to the formulation of public policies on food and nutrition: A scoping review

**DOI:** 10.1371/journal.pgph.0005972

**Published:** 2026-02-23

**Authors:** Ana Carolina Feldenheimer da Silva, Andhressa Araújo Fagundes, Cintia Chaves Curioni, Clara Cecília Ribeiro de Sá, Gabriela Buccini, Maína Ribeiro Pereira-Castro, Mariana Souza Lopes, Luana Lara Rocha, Fernando Marcello Nunes Pereira, Elisabetta Recine

**Affiliations:** 1 Department of Social Nutrition, Rio de Janeiro State University, Rio de Janeiro, Brazil; 2 Postgraduate Program in Nutritional Sciences, Department of Nutrition, Federal University of Sergipe, Aracaju, Brazil; 3 Postgraduate Program in Health Sciences, Universidade Federal de Sergipe, Aracaju, Brazil; 4 Department of Social and Behavioral Health, School of Public Health, University of Nevada Las Vegas, Las Vegas, Nevada, United States of America; 5 Faculty of Education and Health Sciences, University Center of Brasília, Brasília, Federal District, Brazil; 6 Department of Nutrition, Federal University of Paraíba, João Pessoa, Paraíba, Brazil; 7 Department of Preventive and Social Medicine, Federal University of Minas Gerais, Belo Horizonte, Brazil; 8 Department of Nutrition, Faculty of Health Sciences, University of Brasília, Brasília, Federal District, Brazil; World Health Organization Regional Office for South-East Asia, INDIA

## Abstract

This scoping review aims to identify facilitators and barriers to the formulation of public policies on food and nutrition across different contexts. The Scope review was conducted in seven databases. The search strategy was designed using research terms (and their derivatives) and included combinations of ‘nutrition’, ‘food’, ‘policy’, ‘guideline’, ‘action plan’, ‘strategy’, and ‘process’. All stages of data selection and extraction were independently performed by pairs of researchers. A narrative synthesis was developed to present the findings. A total of 17 articles were published in scientific journals, containing data from 28 countries across five continents (Africa, North and South America, Asia, Europe, and Oceania). The studies, published between 1979 and 2023, documented processes for developing policies, programs, and public plans for food and nutrition. The facilitators and barriers described in the policy development process were identified, and they were grouped into seven categories: (1) governance, (2) intersectoral negotiations, (3) political will, (4) policy characteristics, (5) team profile, (6) availability and use of data, and (7) conflict of interest and interference from the private sector. Except for conflict of interest, all categories were pointed out as elements that can potentially facilitate or hinder the policy development process. Understanding the facilitators and barriers to public policy formulation can impact the development process, such as reducing development time or formulating responses that are better aligned with the needs of the population. Policymakers can develop strategies to minimize the impact of barriers and leverage facilitators to develop more efficient policies with better outcomes.

## Introduction

Malnutrition, which encompasses all health outcomes related to undernutrition, weight and height deficits, inadequate intake of macro and micronutrients, overweight, obesity, and chronic non-communicable diseases (NCDs), is a significant global public health challenge [1]. These outcomes are the ones most likely to increase the global burden of disease [2–4]. It is estimated that, in 2022, about 890 million people worldwide were obese and almost 400 million had malnutrition, while 150 million children had height deficit and 45 million had weight deficit [5].

Currently, all countries must deal with at least a combination of malnutrition-related outcomes. In fact, projections for 2030 show that the global prevalence of low-birth-weight newborns, anemia in women aged 15–49 years old, and obesity among adults may affect 14.2%, 32.3% of the world population, and more than 1.2 billion adults, respectively. Such rates are far from reaching the agreed reduction targets [6]. Agendas such as the Sustainable Development Goals (SDGs), in particular SDG 1 – No Poverty, SDG 2 – Zero Hunger and Sustainable Agriculture, and SDG 3 – Good Health and Well-Being, are global initiatives to mitigate the impacts of malnutrition [7].

However, owing to the complexity of these phenomena, the reduction and the ultimate elimination of all forms of malnutrition is still a challenging goal that is far from being achieved by countries. Changing the current scenario requires the development and implementation of effective public policies that can integrate intersectoral measures and address the different determinants of malnutrition [1]. Public policies on food and nutrition play a crucial role in promoting adequate and healthy eating, preventing diseases, and ensuring human rights to adequate food and health [8]. The development of effective policies can potentially impact the health of populations as well as improve economic and social indicators [9].

A few decades ago, different guidelines were published for the formulation of public policies in the field of food and nutrition [10,11]. In the public policy cycle, the policy design process involves several social actors and various interests, and it goes through different stages, for example, raising awareness of realities and challenges, identifying priorities, drawing on successful experiences, evaluating actions that were implemented previously, and ensuring that different sectors are equally considered [12]. Although formal processes have been defined in the public policy cycle, there is little evidence about factors that strengthen and/or undermine the policy development process.

By examining previous experiences and lessons learned, this study seeks to identify patterns and strategies which can inform and guide the formulation of policies that are more effective and tailored to specific needs. In addition, it discusses how different actors - including governments, non-governmental organizations, the private sector, and civil society - interact and collaborate - or not - to formulate policies focused on coping with the main challenges posed by malnutrition. Thus, the objective of this study was to explore facilitators and barriers to the formulation of public policies on food and nutrition focused on responding to the main challenges posed by all forms of malnutrition.

## Method

To ensure a robust and reproducible process, evidence was collected on the process of designing public policies on food and nutrition, based on the guidelines of the Preferred Reporting Items for Systematic Reviews and Meta-Analyses extension for Scoping Reviews (PRISMA-SCR) for reporting results, and according to the methodology proposed by the Joanna Briggs Institute [13]. This review was registered in the Open Science Framework - available at https://osf.io/23yah.

### Research question

The formulation of the research question was based on the Population, Concept, and Context (PCC) framework proposed by the Joanna Briggs Institute [13]: Population (P): Public policies, programs, and plans for food and nutrition in any geographic or institutional context; Concept (C): factors that were considered facilitators and/or barriers during the formulation of public policies, programs and plans for food and nutrition; and Context (C), which explores how these facilitators and barriers affected the process of developing public policies, programs, and plans for food and nutrition. Based on this structure, to address the process of developing public policies, programs and plans for food and nutrition, the following research question has been formulated for the present review: What are the facilitators and barriers to the formulation of public policies, programs, and plans for food and nutrition? Table 1 shows the detailed definitions for the terms used in this review.

**Table 1:** Definition of key concepts included in the study.

Outcome	Definition
Barriers	Structural or procedural barriers that limit the efficiency of government actions, compromising the delivery of public goods and services to society [14].
Facilitators	Favorable conditions that allow the adoption and implementation of evidence-based policies, including strong leadership, political support, adequate resources, and collaboration among the actors involved [15].
Public Policy	Set of guidelines, programs and actions developed by the state to deal with the needs of society. It involves the stages of design, implementation, and evaluation, and may involve different regulatory, financial, and institutional instruments [16–18].
Public plans	Strategic documents/instruments that define medium and long-term objectives, directions, goals, and actions for the development of public policies in a given area. They guide the operationalization of public policies and are usually prepared by government agencies based on technical diagnoses and with social participation [17–19].
Public Programs	Set of concrete actions within a public policy, organized to achieve specific objectives through a budget, deadlines, and previously defined strategies, often involving different governmental agencies and actors. They involve the direct execution of actions in the territory and in a society [17,18].

### Search strategy

A systematic search for published studies was first performed on June 5, 2023 and updated on December 4, 2024, in the online databases MEDLINE, Scopus, Web of Science, EMBASE, CINAHL, and Lilacs. The search strategy was built with search terms (and derivatives) developed from the Medical Subject Headings (MeSH), including combinations of the terms ‘nutrition’, ‘food’, with ‘policy’, ‘guideline’, ‘action plan’, ‘strategy’, and ‘process’. The descriptors and the search strategy used in each database are explained in S1 Text.

Searches were performed without language and date of publication restrictions. All searches in the databases were performed simultaneously. The references were exported to the Zotero reference manager for deletion of duplicates, and they were later transferred to Rayyan for the selection of studies.

### Eligibility criteria

The present study included articles that were published online in peer-reviewed journals, and addressed strategies, methodologies, evaluations of the process of development of food and nutrition policies, reflections, lessons learned, as well as guidelines or conceptual frameworks relative to the formulation of these policies. Studies were excluded if they: (a) did not address the design of food and nutrition policies; (b) evaluated the implementation or impact of policies; (c) compared the content or adequacy of policies and plans with international commitments or guidelines; (d) addressed the development of actions beyond the executive level as laws, regulations, or legislative/judicial projects and voluntary programs; (e) were clinical, intervention or population studies. Despite containing data on public policy formulation, grey literature, including government technical reports, information notes, and documents from international agencies, was excluded from the search. Only studies presenting results or reflections on the public policy formulation process, and published in peer-reviewed journals, with reflections about the formulation process were included.

### Selection of studies

The articles were selected independently by two pairs of reviewers with the help of the Rayyan software. In the first stage, the titles and abstracts were analyzed to verify compliance with the eligibility criteria. In the second stage, the selected articles were read in full. Any disagreements between the reviewers were resolved by bringing in a third reviewer in both stages: abstract selection and full reading of the articles.

### Data extraction

The extracted variables included: country of the study, title, contact email, scope of action development, type of action developed, name of the plan/program/policy, objective of the plan/program/policy, description of the action, facilitators identified by the authors, barriers identified by the authors, actors involved in the process, and the target audience of the action. The data were extracted by two pairs of reviewers, using a standardized data extraction form developed for this purpose in Google Forms. Any disagreements in the extraction of the data were resolved by bringing in a third reviewer.

### Data synthesis

Data synthesis involved the identification and categorization of facilitators and barriers to the development of public food and nutrition policies, with the aim of creating a comprehensive view of the dynamics involved in this process.

The definition of each facilitator/barrier is described in Table 2. The facilitators and barriers were categorized according to the themes found in the studies. Regardless of some studies being multicentered, challenges and barriers were identified only once per study, each observation was considered once on the analysis. No weighting was performed based on the number of countries.

**Table 2:** Definition of categories for classification of facilitators and barriers.

Categories	Definition
Governance	Relationship between institutional structures, relationships between actors and/or organizations, decision-making processes, and incentives. It involves capacity, power, and commitment to act. Multi-sectoral cooperation, vertical coordination and engagement or mobilization of civil society, along with other factors that are necessary for the country to transform policy recommendations into actions [21]
Mechanisms of participation and intersectoral negotiations	Availability of processes and initiatives that allow increased debate with different sectors of civil society, academics, professionals, and those that integrate the social groups directly involved in the agenda in question. Participation may range from consultation to context analysis to planning and monitoring. Intersectoral collaboration is related to processes and strategies of government sectors involved in the theme, other than the sector directly in charge. Intersectoral collaboration implies processes that allow sharing views, objectives, strategies, priorities, and commitments, etc.
Political will	A situation in which a sufficient set of decision-makers, who have a common understanding of a specific problem on the formal agenda, is committed to supporting action and produces a commonly perceived and potentially effective political solution [22]. Degree of commitment among key decision-makers to enact and implement specific policies [23].
Capacity and/or technical profile of the team	Team responsible for the development of a technically qualified policy (document writing, research skills, compilation of data and information) and/or with professional training on food and nutrition initiatives.
Policy characteristics (scope, funding, planning, participation, awareness raising)	Policy-specific elements such as coverage, specific funding, planning actions aimed at policy implementation, participation mechanisms, and policy-related factors that generate policy-related engagement and awareness raising.
Data availability, monitoring, and evaluation	Access to the database, technical reports, and other documents updated with information on nutritional status, food consumption, malnutrition-related disorders, with a view to monitoring and/or evaluating the health and nutrition status of populations, as well as outcomes of interest.
Conflict of interest and interference from the private sector	Situations arising from direct or indirect involvement of individual or collective subjects that interfere with the processes of analysis, discussion, and decision, and compromise the preservation of the common good and public interest.
Other	Impacts of the Covid-19 pandemic, adaptation of international guidelines, and dependence on commercial and scientific experience to shape food and nutrition policies.

After defining the categories of facilitators and barriers, narrative synthesis was performed based on the similarity of themes and then organized according to the nature of information. The reviewers classified the facilitators and barriers inductively by reading the documents in full. Each element was classified by two researchers working separately and independently, and the disagreements were analyzed by two other researchers. Subsequently, these classifications were grouped and categorized. The categories described in Table 2 were defined after the reviewers read all the elements extracted from the articles and grouped them by thematic categories. Each situation extracted from the articles was analyzed by four pairs of researchers and if they had a different understanding during classification, the cases were discussed until the group achieved a consensus. The information extracted was used in the narrative synthesis of the findings. Since this review sought to provide an overview of the policymaking process in the field of food and nutrition, the quality of the studies was not evaluated [20].

## Results

**Fig 1:**
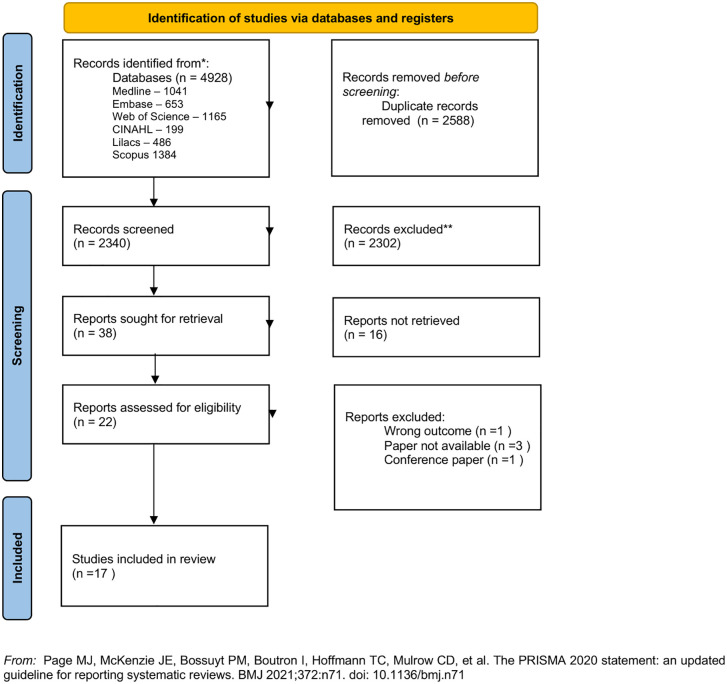
PRISMA-ScR flow diagram of the selection process of the articles included in the review.

The search strategy obtained 4,928 results. After the removal of duplicates (n = 2,588), there were 2,340 single records left, and 38 of them were considered suitable for full text screening in accordance with eligibility criteria. At the end, 17 articles published in peer-reviewed scientific journals were included (Fig 1).

Table 3 shows the scope of policies, programs, and plans evaluated in the manuscripts included in this review. The studies were published between 1979 and 2023, and 58.8% (n = 10) were published in the last 15 years. They discussed processes of development of policies, programs, and plans designed by the government on food and nutrition in 28 countries across the continents: five from Oceania, four from Europe; 3 from North and South America; one from Asia and 3 studies conducted in more than one continent (Asia and Africa; America, Oceania, Europa; America, Europa and Africa). At the jurisdictional level, 64.7% of the manuscripts addressed policies, programs or plans for food and nutrition at the national level (n = 11), and 70.6% referred exclusively to public policies (n = 12).

**Table 3:** Characteristics of the reviewed studies and the scope of the evaluated policies, programs, and plans for food and nutrition.

First Author	Year of publication	Country of Action	Jurisdictional level	Type of Action	Name of Action	Objective of Action	Target audience	Actors involved
Babu	2000	Ghana, India, Kenya, Mali	National	Policy	Credit programs on food security and nutrition in Ghana	–	–	National and international researchers, NGOs, and local managers
Barbour	2023	Australia	Location	Policy	Policies aimed at the promotion of healthy eating and food security	To promote healthy eating and food security at the local level	Resistant population in the Australian state of Victoria	Local managers and civil society involved in policy development
Carey	2015	Australia	National	Plan	Australia’s National Food Plan	–	Australian population	Department of Agriculture, Fisheries and Forestry, Public Health Association of Australia, civil society, and private sector
Chernichovsky	1990	–	–	Policy/Plan	–	–	Food policy makers	Researchers
Gill	2010	Scotland	National	–	Scotland’s national food and drink policy	To support the growth of the food and beverage industry, build the country’s reputation as a land of food and drink, ensure that the population makes healthy and sustainable choices, make the public sector an example of sustainable food procurement, ensure food supplies are safe and resistant to change, make food available and accessible to everyone, and ensure that the population understands more about the foods they eat	–	Federal government sectors, researchers, NGOs, government analysts, farmers, productive sector, and civil society
Iverson	1979	United States	National	Policy	–	To be a tool for problem analysis	Developing countries	Representatives of developed countries, developing countries, international organizations, and researchers
Kapetanaki	2021	Greece	National	Policy	Nutrition policy of Greece	–	Residents of Greece	Government, civil society (consumers, NGOs, scientists, consumer associations) and food supply chain (farmers, manufacturers, retailers, logistics companies, advertising, and food services)
Kjaernes	2008	Finland, Sweden, Norway	National	Policy	Nordic food and nutrition policies	–	Population of the countries involved	Government, nutritionists, and civil society
Latu	2018	Fiji	National	Policy	Seven food-related policies in Fiji	–	Inhabitants of Fiji	Government managers involved in policy formulation
Mannan	2004	United States, Australia, Norway	National	Policy	–	–	Inhabitants of the countries involved	Representatives of the public and private sectors, agriculture, food industry, retail trade, consumers, and the media
Santos	2021	Brazil	National	Policy	National Food and Nutrition Policy (PNAN)	To improve food, nutrition, and health conditions to ensure food and nutrition security to the Brazilian population	Brazilian population	Ministry of Health, Interministerial Chamber for Food and Nutrition Security, National Health Council, National Council of Food Security and Nutrition, researchers, managers, health workers, NGOs, and civil society
Sibbing	2022	United States (Austin, Washington D.C.), Brazil (Belo Horizonte, Curitiba, Rio de Janeiro), United Kingdom (Birmingham), France (Bordeaux), Netherlands (Ede), Belgium (Ghent), Italy (Lucca, Milan), Ecuador (Quito), Canada (Toronto), Namibia (Windhoek)	Municipal	Plan	Milan Urban Food Policy Pact	International agreement on urban food policies signed by more than 200 cities around the world	Urban populations living in the cities that signed the pact	Local managers
Siong	2020	Indonesia, Malaysia, Myanmar, the Philippines, Thailand, Vietnam	National	Plan	National plans of action for nutrition (NPANs)	–	Population of the countries involved	Organizations, donors, and the media
Suárez-Herrera	2009	Canada	–	Program	Community Nutrition Programs in the field of Primary Health care (APS)	Reduction in the incidence of a health problem throughout the population	Collectivity: Individuals, families, and communities	Managers, health workers, politicians, civil society, individuals and families, community groups, small and medium-sized enterprises
Timotijevic	2011	United Kingdom	–	Policy	–	–	Less representative groups (focus on the elderly)	Local managers, health workers, retailers, NGOs, and researchers
Waqa	2017	Fiji	National	Policy	–	–	Policymakers of the Ministry of Health and Medical Services and the Ministry of Agriculture	Managers of the Ministry of Health and the Ministry of Agriculture
Yeatman	2003	Australia	Location	Policy	Food and nutrition policy	To improve the local food system	Resistant population in Australian states: New South Wales, Queensland, and Tasmania	Local managers, health workers, and researchers

The objectives of the most frequently mentioned public food and nutrition policies, programs and plans included healthy eating and food and nutrition security, both at the local and national level, and the quest for a healthier and more sustainable food system (n = 3). Other recurring goals were to strengthen governance and plan food policies, including the creation of tools for problem analysis and adherence to international agreements on urban food policies (n = 3). The articles also mentioned objectives for public health, such as reducing the incidence of health problems in the population, and encouraging healthy and sustainable food choices (n = 2). Finally, some initiatives highlighted the economic development of the food sector, focusing on the growth of the food and beverage industry, the health safety of food products, and the adoption of sustainable procurement practices in public procurement (n = 3).

The audience most frequently cited in public food and nutrition policies, programs and plans was the general population of the countries, states, or cities involved (n = 9), followed by public policymakers (n = 2). The interviewed actors that were most frequently involved in the process of careful consideration during the development of these initiatives, were local managers (n = 6), civil society (n = 5), researchers (n = 5), non-governmental organizations (NGOs) (n = 5), and government sectors such as health and agriculture (officers in federal departments, ministers, and government managers) (n = 5).

**Fig 2:**
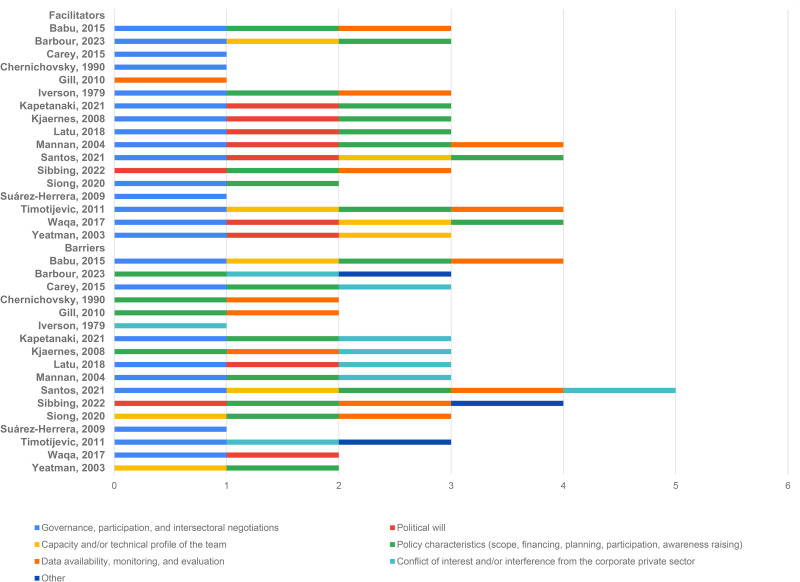
Categorization of facilitators and barriers identified by studies that described the processes of design of policies, programs, and plans for food and nutrition.

Fig 2 shows a systematization of facilitators and barriers often found while designing public policies, programs, and plans for food and nutrition. Importantly, except for “conflict of interest” and “interference from the private sector”, the other categories of facilitators and barriers have a dual characteristic, that is, they may apply to either one of the categories depending on the situation.

The most frequent facilitators were governance, participation mechanisms, and intersectoral negotiations (88.2%, n = 15), which includes, for example, social participation, consultation with researchers for the formulation of initiatives, connection with international leaders or agencies, creation of advisory council, and conflict resolution through negotiation. Next, policy characteristics (scope, funding, planning, participation, awareness raising) was the second most identified facilitator (70.6%, n = 12), which includes recognition of cultural aspects, alignment of state and federal policies on the agenda of action, recognition of nutrition as a social problem, definition of public budget, use of scientific evidence, and carefully considering the needs and priorities of the population. Political will (47.1%, n = 8), availability of data for situational and/or nutritional diagnosis, monitoring, and evaluation (35.3%, n = 6), and capacity and/or technical profile of the team (29.4%, n = 5) were also identified as facilitators. Table 4 shows a detailed description of these data.

**Table 4:** Description of facilitators and barriers identified by studies that described the processes of formulation of policies, programs and plans for food and nutrition.

Identification of the article	Facilitators identified	Description of facilitators	Barriers identified	Description of barriers
Babu, 2000	Governance, participation, and intersectoral negotiations	Intersectoral negotiations are recognized as weak Stronger collaborative capacity of technical staff of the Ministries to use information to generate indicators Participation of social control in some stages of the process Frequent feedback from staff to policymakers Frequent consultation with researchers and technical team of the government during the phase of analysis	Governance, participation, and intersectoral negotiations	Representatives of the same agency or office, with different institutional status, with different views on the policy, working in the negotiation phases Lack of intersectoral discussions Lack of active engagement of all participants in the discussion
	Availability of data/information	Identification of indicators (quantitative and qualitative) that could be created and monitored, based on the available information that can be used to identify the most vulnerable groups	Capacity and/or technical profile of the team	Intergenerational disagreement among managers (young people did not feel comfortable to speak in front of older people)
	Policy characteristics (scope, funding, planning, participation, awareness raising)	To recognize cultural and circumstantial issues, such as different gender roles, food choices, family spending patterns	Policy characteristics (scope, financing, planning, participation, awareness raising)	Lack of recognition of the risk of designing results-based policies without proper understanding of the underlying processes Lack of understanding of all elements required for determining nutritional status Failure to identify confounding factors that can help direct policies correctly Unequal participation of sectors in the process
			Data availability, monitoring, and evaluation	Collection of a large set of information that was not reverted into indicators Failure to address issues such as the cost and effectiveness of programs
Barbour, 2023	Capacity and/or technical profile of the team	Team is committed and focused on develop policies Leaders are committed and competent and take responsibility for the agenda	Policy characteristics (scope, funding, planning, participation, awareness raising)	Insufficient financial resources, which was interpreted as low commitment of the other spheres of government to local policies Misalignment of state and federal policies, generating conflict in the execution of the agenda, with interruption of funding and lack of response to local needs
	Governance, participation, and intersectoral negotiations	Engaged community: previous existence of organized groups and establishment of attentive listening and participation mechanisms to identify and respond to population demands Partnerships with community groups, local organizations, neighboring communities, and state managers who have strategic relationships with the agenda Connections with global organizations and leaders (e.g., Unicef)	Conflict of interest and/or interference from the corporate private sector	Conflicting interests between decision-makers from different spheres and between managers and the community
	Policy characteristics (scope, funding, planning, participation, awareness raising)	Existing state and federal policies aligned with the agenda	Other	Impacts of the COVID-19 pandemic: social distancing demobilized collectives, prevented face-to-face meetings, hindered the participation of some groups in the discussions, generated important economic impacts in community groups
Carey, 2015	Governance, participation, and intersectoral negotiations	Support and involvement of the general population Development of strong intersectoral alliances with civil society groups that enable greater political leverage (in the areas of environment, social justice, and community food sectors)	Governance, participation, and intersectoral negotiations	The lack of transparency in the activities of the Working Group, the agendas and minutes of the meetings were not publicized Critical sectors were excluded from the agendas of the policy (academics, civil society, and stakeholders in public health) Sub-representation of sectors in the nation-wide debate
			Policy characteristics (scope, funding, planning, participation, awareness raising)	Marginalization of public health, nutrition, and environmental sustainability
			Conflict of interest and interference from the private sector	Strong participation of the food industry and government sectors associated with it Sectors such as health, consumers, and environmental advocates were prevented from participating in the political forums Thematic document emphasized the maximization of food production, promoting a competitive, productive environment and defending an efficient food industry
Chernichovsky, 1990	Governance, participation, and intersectoral negotiations	Social participation	Policy characteristics (scope, funding, planning, participation, awareness raising)	Misuse of government funds
			Data availability, monitoring, and evaluation.	Lack of information on the target group of the program Lack of awareness of the nutritional status of the population
Gill, 2010	Data availability, monitoring, and evaluation.	Long-term investment in research to generate evidence that supports policies	Policy characteristics (scope, funding, planning, participation, awareness raising)	Language barriers and cultural differences
			Data availability, monitoring, and evaluation.	Unavailable evidence on industry interests, hindering the decision-making process Lack of communication about the complexities and uncertainties of planned actions, not adequately informing policymakers and the population at large
Iverson, 1979	Governance, participation, and intersectoral negotiations	To determine areas of consensus To show the different positions defended in areas of controversy To identify the arguments in favor of and against the policy To develop effective administration	Conflict of interest and/or interference from the corporate private sector	Conflicting views
	Data availability, monitoring, and evaluation.	To understand the context of the problem To develop evaluation criteria		
	Policy characteristics (scope, funding, planning, participation, awareness raising)	To develop relevant operational objectives		
Kapetanaki, 2021	Governance, participation, and intersectoral negotiations	The interest of the population in the development of policies In cases of asymmetry of power, there is a need to ensure cooperation and trust Definition of shared goals throughout the food system Existence of a model that focuses on the collaboration between interdependent actors with the common goal of citizenship and well-being	Governance, participation, and intersectoral negotiations	Power imbalance and conflicting objectives among actors Weak government Lack of trust between sectors Interdependence and power asymmetry
	Political will	Active government involved in policy development	Policy characteristics (scope, funding, planning, participation, awareness raising)	Clear need for a focus on citizens
	Policy characteristics (scope, funding, planning, participation, awareness raising)	Development of marketing concept to make the population understand the objectives of the policy and acknowledge the value of its scope	Conflict of interest and/or interference from the corporate private sector	Fundamental differences of interest Strong lobbying power of large food corporations
Kjaernes, 2008	Governance, participation, and intersectoral negotiations	Local efforts and decentralization Formulation of advisory council Production and use of evidence, networking, advocacy, and defense in the development of policies in each country	Policy characteristics (scope, funding, planning, participation, awareness raising)	Competition between themes (e.g., nutrition vs. agriculture)
	Political will	The authorities recognize the nutrition agenda as their responsibility Institutionalization of the field Political will	Data availability, monitoring, and evaluation.	Specific evaluation of programs that made little contribution to the policy
	Policy characteristics (scope, funding, planning, participation, awareness raising)	Understanding nutrition as a social problem	Conflict of interest and/or interference from the corporate private sector	Conflicts with agricultural sectors Use of the principle of ‘ informed autonomy’ to confuse the population The conflict between interests of a neoliberal state and government investments in health
Latu, 2018	Governance, participation, and intersectoral negotiations	Social participation Successful collaborations at different stages of policy development	Governance, participation, and intersectoral negotiations	Lack of consultation with relevant stakeholders Change and priority deviation
	Political will	Leaders’ commitment to carry out policies, combined with governmental support at the time of deliberation The political environment of the time	Political will	Poor leadership due to lack of motivation by government policymakers to engage in early stages and during the development of policies Leaders do not support policy progress
	Policy characteristics (scope, funding, planning, participation, awareness raising)	The nature of policies	Conflict of interest and interference from the private sector	Strong influence of the food industry on government policymakers
Mannan, 2004	Governance, participation, and intersectoral negotiations	Consensus and multisectoral involvement, with coordinated effort and joint establishment of a National Nutrition Council in 1937 Single coordination between sectors Conflict resolution through negotiation	Governance, participation, and intersectoral negotiations	Power dispute and competition between sectors of the same government (e.g., agriculture and health competed for the leading role in formulating and implementing the food and nutrition policy in Norway)
	Political will	Strong commitment of the government to improve the nutritional and health status of the population Theme present on the political agenda of the Federal Government	Policy characteristics (scope, funding, planning, participation, awareness raising)	Difficulty in integrating nutritional issues into ongoing institutional mechanisms and procedures; Lack of alignment between the objectives of the country’s nutrition and economic policies.
	Data availability, monitoring, and evaluation.	To have an Inter-agency Council for monitoring nutrition actions	Conflict of interest and/or interference from the corporate private sector	Conflict of interest between policymakers (processed food manufacturers go against healthy eating guidelines Industry’s lobbying causes conflicts in decision-making Different interests between sectors (economic interests vs population health, risk reduction vs costs)
	Policy characteristics (scope, funding, planning, participation, awareness raising)	Nutritional guidelines aimed at young people Nutrition research to develop comprehensive work plans and specific government budgets for nutrition actions National guidelines developed for food supply and nutrition policy to promote public health and increase agricultural production		
Santos, 2021	Governance, participation, and intersectoral negotiations	The intersectoral nature of the policy Negotiations between different systems (Health and Food and Nutrition Security), in addition to interaction with other governmental sectors International partnerships to evaluate specific programs	Governance, participation, and intersectoral negotiations	Absence of adequate physical structure to implement actions;
	Political will	Presence of the theme and policy in official instruments of government planning	Capacity and/or technical profile of the team	Low capacity for management, planning and evaluation of food and nutrition actions; Need to qualify professionals who will implement the policy;
	Capacity and/or technical profile of the team	Professional profile of policy managers (nutritionist) Qualified technical staff to develop actions	Policy characteristics (scope, funding, planning, participation, awareness raising)	Low coverage and use of the health system;
	Policy characteristics (scope, funding, planning, participation, awareness raising)	Policy actions were included in official instruments, such as national plans Existence of financial resources for food and nutrition actions at the federal, state, and municipal levels	Data availability, monitoring, and evaluation.	Information base is inconsistent for decision-making
			Conflict of interest and/or interference from the corporate private sector	The food industry uses strategies to delay and/or prevent the approval of the policy.
Sibbing, 2022	Political will	To include/Maintain the theme on the political agenda To increase awareness of food policies	Political will	Lack of commitment and collaboration of actors in the process, including government officials;
	Data availability, monitoring, and evaluation.	Design of a framework of indicators has enabled the achievement of political priorities and objectives Identification of available data and gaps to advance monitoring and evaluation Evidence generation through continuous monitoring To identify priorities and to achieve political objectives through involvement in monitoring and evaluation To use monitoring and evaluation to strengthen connections between different departments and/or stakeholders	Policy characteristics (scope, funding, planning, participation, awareness raising)	Lack of enough financial resources, capacity, knowledge, or organizational infrastructure for evaluation
	Policy characteristics (scope, funding, planning, participation, awareness raising)	Generate political will using evidence Clearly define actions for a city’s food policy	Data availability, monitoring, and evaluation.	Insufficient and low-quality data Government data is inaccessible, which results in dependence on several sectors to obtain information Low priority/acknowledgment of the value of monitoring and evaluation actions Difficulty in evaluating results or impact Failure to organize continuous and sustainable evaluation cycles
			Other	Adaptation of international guidelines to local reality;
Siong, 2020	Governance, participation, and intersectoral negotiations	Participation of civil society Establishing specific goals based on the country’s need and according to global nutrition goals Support from more developed countries	Capacity and/or technical profile of the team	Need for greater coordination among stakeholders Lack of implementation capacity
	Policy characteristics (scope, funding, planning, participation, awareness raising)	Government funding	Policy characteristics (scope, funding, planning, participation, awareness raising)	The government’s lack of significant financial commitment to policies
			Data availability, monitoring, and evaluation.	Monitoring and improvement of the evaluation system
Suárez-Herrera, 2009	Governance, participation, and intersectoral negotiations	Social participation	Governance, participation, and intersectoral negotiations	Deregulation of the spaces of social participation, in which the collective interests of highly organized groups overlap the specificities and needs of the local population.
Timotijevic, 2011	Governance, participation, and intersectoral negotiations	Reform in the governance of food and nutrition policy for greater involvement of the public Simultaneous implementation of different forms of representativeness	Governance, participation, and intersectoral negotiations	Self-representation in policy-building spaces Only of scientific evidence is considered important while public opinion is disregarded Exclusion of citizens from food-related political decisions
	Data availability, monitoring, and evaluation.	Use of evidence to develop policies	Conflict of interest and/or interference from the corporate private sector	Dependence on commercial and scientific experience to shape food and nutrition policies owing to the lack of full consideration of consumer concerns
	Capacity and/or technical profile of the team	To form technical groups with a diversity of technical expertise and considering “expertise by experience”.		
	Policy characteristics (scope, funding, planning, participation, awareness raising)	To create a department for communication with the population to include the public that traditionally was less likely to be represented in policy development		
Waqa, 2017	Governance, participation, and intersectoral negotiations	To leverage an inter-ministerial agenda To ensure stakeholder engagement in the process to increase trust	Governance, participation, and intersectoral negotiations	Increased government political influence over Ministry decreases participation of some non-governmental sectors The lack of inter-ministerial collaboration has had a negative impact on political support and operationalization of policies Staff turnover Bureaucracy
	Political will	Prioritization of policy inclusion in planning	Political will	Lack of political will, poor understanding of commercial policies, competing governmental priorities, and low level of awareness about the problem No acceptance of proposals by managers
	Capacity and/or technical profile of the team	Training in policy development and research skills Employee competence		
	Policy characteristics (scope, funding, planning, participation, awareness raising)	To start the process with meaningful conversations to ensure that policies reflect individual needs and are relevant Engagement with stakeholders increased demand for evidence and use of them, resulting in increased awareness of the problem and, consequently, greater motivation to develop and implement policies		
Yeatman, 2003	Governance, participation, and intersectoral negotiations	Partnerships	Capacity and/or technical profile of the team	To develop this knowledge and skills related to the political process and the ability to recognize opportunities and to be able to apply them
	Political will	Creation of political committees	Policy characteristics (scope, funding, planning, participation, awareness raising)	Funding Food environment
	Capacity and/or technical profile of the team	Research and report writing skills		

The most frequent barrier was related to policy characteristics (scope, funding, planning, participation, awareness raising) (70.6%, n = 12); it includes no prevision of financial resources for policy implementation, corruption involving the public budget, cultural and linguistic diversity in the scope of the initiative, which may cause barriers such as low coverage and use of the health system, low priority for the nutrition and public health agenda, and previous laws and regulations may have objectives that conflict with the policy under development. For example, a national law permitting pesticide use may contradict local community efforts to implement organic farming, highlighting the need to address such inconsistencies in the policy formulation process. The second most frequently mentioned barrier was governance, participation, and intersectoral negotiations (52.9%, n = 9), which includes, for example, insufficient discussions and intersectoral negotiations, lack of trust across sectors, lack of transparency in the activities and stages of the design process, interdependence and asymmetry of power, and lack of consultation with stakeholders.

Facilitators were the availability of data for situational diagnosis, monitoring, and evaluation (41.2%, n = 7), capacity and/or technical profile of the team (23.5%, n = 4), and political will (17.6%, n = 3). Conflict of interest and interference from the private sector (47.1%, n = 8) stood out among barriers, which also include conflicting interests among stakeholders, strong participation of the food processing industry in discussions on initiatives, strong lobbying power of large food corporations, and action of the food industry to delay or prevent the approval of the policy. The “Other” category was also included when characterizing barriers (17.6%, n = 3), and it covers impacts of the Covid-19 pandemic, adaptation of international guidelines to the local reality, and dependence on commercial and scientific experience to shape food and nutrition policies.

## Discussion

The design of management tools, whether they are plans, policies or programs, are crucial to responding to the challenges of the dual burden of malnutrition. The process of designing these instruments involves a range of factors and actors and the presence or absence of these elements can facilitate or hinder this process. This study analyzed factors that were considered facilitators and/or barriers to the process of developing policies, plans, and programs.

The results were grouped into seven groups: policy characteristics (scope, funding, planning, participation, awareness raising); governance; intersectoral participation and negotiations; political will; capacity and/or technical profile of the team; data availability, monitoring, and evaluation; conflict of interest; and interference from the private sector. Facilitators and barriers were consistently identified across countries, irrespective of their political structures or geographical locations. The same factor can be considered either a facilitator or a barrier, depending on how its presence or absence interferes with the policy development process. An example is the participation of the local community in the process of formulating initiatives, which was identified as a facilitator and a barrier by Suarez-Herrera, Juan & Serra-Majem [24].

The essential characteristics for the operation of a policy are determined by the process of designing and planning it during the subsequent phases of the public policy cycle, considering the aspects that underpinned the design of such policy. A policy is structured in response to the context, considering cultural and circumstantial issues, such as gender roles, family spending patterns, and food choices [25]. Therefore, scientific evidence-based grounds and the availability of an adequate budget are crucial aspects for effective food and nutrition policies [1,12].

Santos et al. [26] and Siong et al. [27] pointed out that budget forecasting for the implementation of food and nutrition policies is one of the main aspects for implementation. Sibbing et al. [28] and Santos et al. [26] argued that the allocation of resources requires well-defined and planned actions on the government agenda. These characteristics are facilitating elements found as early as in the design stage. On the other hand, Barbour, Woods & Brimblecombe [29] pointed out that the lack of funding and/or prioritization on the political agenda can hinder this process. According to this author, another relevant barrier is the misalignment between food and nutrition policies at the different levels of management (state and federal), which can negatively affect the allocation of funds to policies.

Despite the relevance of budget (un)availability for food and nutrition policies since the design stage, this is not the only element that deserves attention. Although the starting point is to understand that food and nutrition are social issues, the participation of civil society and intersectoral negotiations are also pointed out as relevant and facilitators of this stage. In this sense, channels for direct communication with the population are needed to turn a policy, its objectives, and scope into a broad demand that, if met, can satisfy real needs [12,30–33].

While the engagement of government sectors and society in the process is considered a positive distinguishing feature, unequal participation among sectors in the process is highlighted as a barrier to policy formulation [25]. Moreover, competition between themes - which encompasses sectors and areas - is also a relevant aspect [30]. Power dispute between government areas such as health and agriculture [34], and political interference on technical bodies [33] can compromise the coherence of food and nutrition policies and programs. According to Kapetanaki, Tzempelikos & Halliday [31], power imbalance and lack of confidence among sectors can create significant barriers. Structural and political challenges can compromise the effectiveness of governance in the field of food and nutrition. Carey [35] and Timotijevic, Barnett & Raats [32] argued that the lack of accountability and the exclusion of strategic sectors make it difficult to design policies that are aligned with the needs of the population.

Evidence indicates that intersectoral collaboration in public policies, for example, food and nutrition ones, can potentially generate effective and sustainable outcomes while ensuring rights to the population, but it can also pose new challenges [36].

In this scenario of potentialities and challenges of intersectoral collaboration, it is noteworthy that intersectoral negotiations and education and training of technical staff at the governmental level can be fundamental strategies to improve governance in this field [25,35]. Kettl [21] pointed out that governance characterizes the way in which decision-making processes and interactions between governmental and non-governmental actors are organized for the design and implementation of public policies. In the process of structuring food and nutrition policies and programs, governance manifests itself through the coordination of intersectoral networks, regulation of interests, and creation of institutional mechanisms to ensure rights. Therefore, governance expresses the state’s capacity to structure policies and foster cooperation between different levels of government and social actors, according to Pierre and Peters [37]. Furthermore, Barbour, Woods & Brimblecombe [29] and Kapetanaki, Tzempelikos & Halliday [31] emphasized that social participation and the definition of shared objectives are crucial to enable effective collaborative models, especially in policies that bring together multiple sectors, such as health, social assistance, education, and agriculture.

Owing to the complex nature of the food and nutrition agenda and, consequently, the set of actions required for sustainable outcomes, different segments of society are expected to carefully consider their needs and priorities, and different sectors of government are supposed to commit to actions, allocate resources, and achieve goals [38]. Different forms of social participation tend to have a positive impact on the effectiveness of public policies by ensuring that governments taken real demands into account [39].

However, both participation and intersectoral collaboration remain as challenges in the public policy cycle, and governance structures do not foresee or delimit participation. Lack of investment in legitimate and effective processes of participation and intersectoral collaboration poses challenges that increase the impossibility of accessing different perspectives on reality and making detailed descriptions of determinants, nuances, values, and demands. These aspects can lead to bureaucratization of the process of conceiving and implementing public policies, which, in turn, may compromise results and impacts [40]. Similarly, processes that do not form partnerships or encourage actual commitment can also compromise results. Despite specific responsibilities and capabilities, all major issues nowadays demand shared commitment if they are to be addressed and solved. Intersectoral collaboration allows a more integrated and efficient approach to public policies, avoiding overlaps and gaps, and it demands a well-structured governance model.

In addition to policy characteristics, governance, and social participation, conflict of interest and interference from the industry pose significant challenges, emerging as a barrier to the design of food and nutrition public policies, and often compromising the implementation of effective regulations, transparency, and accountability of public authorities. The strong presence of the ultra-processed food industry in decision-making spaces, lobbying by large corporations, and policy capturing on the grounds of commercial interests are recurrent facts that compromise the formulation of strategies aimed at the public interest [41–43]. The studies included in this review indicate that this influence manifests itself both through the direct participation of the industry in boards and committees and through the dissemination of narratives that weaken regulatory measures, such as the taxation of sugary drinks and front-of-pack labeling [26,29,30,31,34,35,44,45]. Industrial activity is already known for its attempt to interfere with public policies, and its tactics have already been mapped out and categorized as corporate political activities (CPAs). CPAs are a series of coordinated and sophisticated strategies to protect business interests, including intimidation and defamation of critics, delegitimization of science, influence on the political process, and creation of corporate alternatives to public policies, such as voluntary programs instead of structured public policies [46].

To minimize this interference, it is essential to establish codes of conduct, strengthen mechanisms of social participation and supervision, invest in multi-institutional coordination, foster collaboration networks, and promote education and awareness of conflict of interest. These actions are fundamental to ensure that the design of public policies is grounded on scientific evidence and the human right to adequate food, without undue influence from the private sector [46,47].

Another element pointed out as important for the policy formulation process was political will, which can be understood as a political scenario in which political institutions, social norms, the historical context, and the informational environment are aligned, and the authorities with decision-making power decide to put the process in place [22]. The presence or lack of political will interfere with policy development. Some situations were seen as facilitators related to political will, namely when authorities engaged and were committed to the nutrition agenda [30,34,45], and supported the whole process; technical areas were institutionalized; the nutrition agenda was on the political agenda and in official documents [26,28,31,33]; and political committees were created [48]. This perception was found in eight studies [26,28,30,31,33,34,45,48].

Political will was understood as a barrier when policymakers were weak leaders or did not support the progress of action, for example, when there was a lack of consultation with relevant stakeholders [45], lack of commitment and collaboration of actors in processes including government officials [28] and competing government priorities [33]. It was also seen as a barrier when decision makers were not committed enough, when the nutrition agenda was not among government priorities, and when managers did not accept the proposed action. Shen [23] stated that the greater the political will, the more likely policies are to be innovative, and the more likely those policies are to be institutionalized.

In the logic of political will, leadership plays a central role in this process, with a direct influence on the formulation, implementation, and evaluation of policies. Santos [26] highlighted that leadership positions should be occupied by nutritionists with expertise in public health to counteract a history of professionals from other fields holding management positions. The former profile favors the sensitivity required to deal with the cultural, social, and technical specificities of food and nutrition in different contexts. Complementing this perspective, Barbour, Woods & Brimblecombe [29] pointed out that effective leadership is one that proposes innovations, stimulates evidence-based decision-making, encourages exchange of experiences, and is aligned with the political agenda in a committed, competent, and responsible manner. Developing and strengthening leadership capacities in food and nutrition are strategic measures to help professionals to be creative, bold, and able to leverage their teams, working not only at the community level, but also at the political level [49].

According to Barbour, Woods & Brimblecombe [29], Timotijevic, Barnett & Raats [32] and Yeatman [48], another key factor for facilitating the organization of food and nutrition policies is perseverance, which can be defined as the commitment and focus required for framing sustainable public policies. When it comes to the composition of work teams, varying types of technical profiles with different backgrounds and relevant professional history can contribute to the process. In addition, continuous training of staff in policy development, combined with research and reporting skills, strengthens the institutional capacity to respond to society’s demands.

However, some important barriers need to be overcome to advance the design of food and nutrition policies. For example, Babu [25] pointed to intergenerational differences between managers, and noted that young people often do not feel comfortable about expressing their ideas in the presence of more experienced managers. In addition, there is a need for greater coordination in working processes among stakeholders [27], which requires combined efforts and continuous dialogue. Implementation capacity also appears as a central challenge, as it requires technical and institutional preparedness to transform plans into concrete actions. According to Yeatman [48], professionals need to develop knowledge and skills related to the political process, as well as an ability to recognize opportunities, so that they can apply them strategically and effectively.

In this process of recognition of opportunities for the sake of policymaking, the use of evidence is a fundamental aspect. The following facilitators stand out: identification of indicators to monitor actions and identify the most vulnerable groups; team with expertise in data processing; and investment in research to generate evidence that can support policies. Two examples of successful experiences in the design of food and nutrition policies are the creation of an Inter-agency Council to monitor food and nutrition initiatives [30,34], and the design of a framework of indicators using available data and gaps to provide opportunities for the achievement of political priorities and progress in monitoring and evaluation [28].

On the other hand, the lack of information was pointed out as a barrier. To change this scenario, here are some possible actions: acknowledging the importance of monitoring and evaluation activities by proposing continuous and sustainable evaluation cycles; improving the quality of the data; evaluating specific programs to support the overall evaluation of policies; and optimizing the information collected to generate more effective indicators. Data availability is fundamental to design evidence-based public policies, whether sectoral or intersectoral, universal or focused, emergency or structuring, of any area. Using reliable data increases the opportunity to develop more appropriate and effective policies based on reality; to identify and prioritize health problems; and to monitor and evaluate the impact of actions. They serve as a guiding principle for governance and help to ensure that the population can access their rights [50].

It is important to have a reliable and qualified information base about the territory, population profile, and health history to direct and sustain decision-making. Villela and Gomes [50] argued that “the identification of the determinants of the health-disease process, of health inequalities, and the impact of actions and programs to reduce the burden of diseases on the population is only possible when using good information at the appropriate time” [our translation]. The effort to ensure reliable and relevant data - both from the routine of services and from population surveys - organizes the work process, directs actions, and optimizes resources and work for priority and necessary issues, resulting in more effective public policies.

The design of public policies is an important step in addressing the causes and consequences of malnutrition. Understanding and summarizing the factors that interfere positively or negatively in this process has the potential to support and enable the design of effective public policies, helping to prevent stress and rework in this process, in addition to ensuring more efficient processes.

## Final remarks

A greater understanding of the aspects and characteristics that facilitate or challenge the design of policies and plans for food and nutrition can enhance the commitment of the public power to this agenda as well as increase the demand from society for more effective actions. In practice, identifying facilitators and barriers is a crucial tool during the planning stage. Policymakers should acknowledge the interconnection among these seven categories. Conducting a thorough diagnosis in each context is essential for developing effective strategies to address barriers and enhance facilitators, which may differ across various settings. In addition to the broad and historically recognized food and nutritional issues that demand government action, new challenges are added to this agenda; for example, the complex relationship between food issues and climate change that reinforce the need to reduce the deficit of public policies on food and nutrition.

## Supporting information

S1 Text Search strategies used in online databases to identify eligible studies. (DOCX)

S1 Checklist PRISMA-ScR checklist. (PDF)

## References

[1] Swinburn BA, Kraak VI, Allender S, Atkins VJ, Baker PI, Bogard JR, et al. The Global Syndemic of Obesity, Undernutrition, and Climate Change: The Lancet Commission report. Lancet. 2019;393(10173):791–846. doi: 10.1016/S0140-6736(18)32822-8 30700377

[2] Askari M, Heshmati J, Shahinfar H, Tripathi N, Daneshzad E. Ultra-processed food and the risk of overweight and obesity: a systematic review and meta-analysis of observational studies. Int J Obes (Lond). 2020;44(10):2080–91. doi: 10.1038/s41366-020-00650-z 32796919

[3] Bortolini GA, de Oliveira TFV, da Silva SA, Santin RDC, de Medeiros OL, Spaniol AM, et al. Feeding and nutrition efforts in the context of primary healthcare in BrazilMedidas relativas a la alimentación y la nutrición en la atención primaria de salud en Brasil. Rev Panam Salud Publica. 2020;44:e39. doi: 10.26633/RPSP.2020.39 32355501 PMC7189826

[4] Suksatan W, Moradi S, Naeini F, Bagheri R, Mohammadi H, Talebi S, et al. Ultra-processed food consumption and adult mortality risk: a systematic review and dose-response meta-analysis of 207,291 participants. Nutrients. 2021;14(1):174. doi: 10.3390/nu14010174 35011048 PMC8747520

[5] WHO. Malnutrition. 2024. Available from: https://www.who.int/news-room/fact-sheets/detail/malnutrition

[6] FAO, IFAD, UNICEF, WFP, WHO. The State of Food Security and Nutrition in the World 2024 – Financing to end hunger, food insecurity and malnutrition in all its forms. Rome. 2024. Available from: doi: 10.4060/cd1254en

[7] WHO. Global action plan for the prevention and control of noncommunicable diseases 2013-2020. Geneva: WHO; 2013. https://iris.who.int/bitstream/handle/10665/94384/9789241506236_eng.pdf?sequence=1

[8] de Castro IRR. Malnutrition, inequity and the guarantee of the human right to adequate food. Cien Saude Colet. 2019;24(7):2376. doi: 10.1590/1413-81232018247.15392019 31340256

[9] Fagundes AA, Damião JDJ, Ribeiro RCL. Reflexões sobre os processos de descentralização da Política Nacional de Alimentação e Nutrição nos seus 20 anos. Cad Saude Publica. 2021;37(Suppl 1):e00038421. 10.1590/0102-311X00038421 34730724

[10] Brasil, Ministério da Saúde, Secretaria de Atenção à Saúde, Departamento de Atenção Básica. Política Nacional de Alimentação e Nutrição. Brasília: Ministério da Saúde. 2013.

[11] FAO. Voluntary Guidelines to support the progressive realization of the right to adequate food in the context of national food security. 2015. [cited 2024 Jun]. Available from: https://openknowledge.fao.org/server/api/core/bitstreams/ed136ed7-338c-4cca-8e43-476b2c3c89f4/content

[12] Souza C. Coordenação de políticas públicas. Brasília: Enap; 2018.

[13] Peters MD, Godfrey C, McInerney P, Munn Z, Tricco AC, Khalil H. Scoping reviews. In: Aromataris E, Lockwood C, Porritt K, Pilla B, Jordan Z, editors. BI Manual for Evidence Synthesis. JBI; 2024. doi: 10.46658/jbimes-24-09

[14] Bardach E. A Practical Guide for Policy Analysis: The Eightfold Path to More Effective Problem Solving. CQ Press; 2012.

[15] Nutley S, Walter I, Davies H. Using evidence: How research can inform public services. Policy Press; 2007.

[16] Howlett M, Ramesh M, Perl A. Studying public policy: Policy cycles and policy subsystems. Oxford University Press; 2020.

[17] Heringer FRA. Quantas políticas públicas há no Brasil? O problema da imprecisão conceitual para a avaliação de políticas públicas. Brasília. 2018. pp. 73.

[18] Wu X, Ramesh M, Howlett M, Fritzen S. Guia de políticas públicas: gerenciando processos; translated by Ricardo Avelar de Souza. Brasilia-DF: Escola Nacional de Administração Pública (ENAP). 2014. pp. 160.

[19] Brasil. Avaliação de políticas públicas: guia prático de análise ex ante I. Brasília-DF: IPEA. V. 1. pp. 192. Available from: https://repositorio.ipea.gov.br/bitstream/11058/8285/1/Avaliacao_de_politicas_publicas_guia_pratico_de_analise_%20ex_ante.pdf

[20] Page MJ, McKenzie JE, Bossuyt PM, Boutron I, Hoffmann TC, Mulrow CD, et al. The PRISMA 2020 statement: an updated guideline for reporting systematic reviews. BMJ. 2021;372:n71. doi: 10.1136/bmj.n71 33782057 PMC8005924

[21] Kettl DF. The transformation of governance: public administration for twenty-first century America. Baltimore: Johns Hopkins University Press; 2002.

[22] Post LA, Raile ANW, Raile ED. Defining political will. Polit Policy. 2010;38(4):653–76. doi: 10.1111/j.1747-1346.2010.00253.x

[23] Shen SV. Political will as a source of policy innovation. Policy Stud J. 2024;53(1):185–200. doi: 10.1111/psj.12571

[24] Suárez-Herrera JC, O’Shanahan JJJ, Serra-Majem L. La participación social como estrategia central de la nutrición comunitaria para afrontar los retos asociados a la transición nutricional. Rev Esp Salud Publica. 2009;83(6):791–803. doi: 10.1590/s1135-57272009000600004 20111828

[25] Babu SC, Brown LR, McClafferty B. Systematic client consultation in development: the case of food policy research in Ghana, India, Kenya and Mali. World Dev. 2000;28(1):99–110. doi: 10.1016/s0305-750x(99)00110-2

[26] Santos SMCD, Ramos FP, de Medeiros MAT, da Mata MM, de Vasconcelos FDAG. Advances and setbacks in the 20 years of the Brazilian National Food and Nutrition Policy. Cad Saude Publica. 2021;37Suppl 1(Suppl 1):e00150220. doi: 10.1590/0102-311X00150220 34730732

[27] Siong TE, Florentino RF, Hardinsyah H, Noor IM, Hlaing LM, Chotivichien S, Hop S. A review of national plans of action for nutrition in Southeast Asian countries. Mal J Nutr. 2020;26(3):501–24. doi: 10.31246/mjn-review-26-3

[28] Sibbing LV, Duncan J, Arcuri S, Galli F, Bock BB. Assessing what food policies lead to on the ground: exploring opportunities and challenges of the MUFPP indicator framework. Agroecol Sustain Food Syst. 2022;46(9):1414–39. doi: 10.1080/21683565.2022.2106007

[29] Barbour LR, Woods JL, Brimblecombe JK. Perseverance, partnerships and passion: ingredients for successful local government policy to promote healthy and sustainable diets. BMC Public Health. 2023;23(1):1762. doi: 10.1186/s12889-023-16656-x 37697341 PMC10494407

[30] Kjaernes U. Food and nutrition policies of Nordic countries: how have they been developed and what evidence substantiates the development of these policies? Proc Nutr Soc. 2003;62(2):563–70. doi: 10.1079/pns2003269 14506905

[31] Kapetanaki AB, Tzempelikos N, Halliday SV. Building relationships: Is this the answer to effective nutrition policy formulation? J Consum Aff. 2021;55(3):1090–110. doi: 10.1111/joca.12396

[32] Timotijevic L, Barnett J, Raats MM. Engagement, representativeness and legitimacy in the development of food and nutrition policy. Food Policy. 2011;36(4):490–8. doi: 10.1016/j.foodpol.2011.04.005

[33] Waqa G, Moodie M, Snowdon W, Latu C, Coriakula J, Allender S, et al. Exploring the dynamics of food-related policymaking processes and evidence use in Fiji using systems thinking. Health Res Policy Syst. 2017;15(1):74. doi: 10.1186/s12961-017-0240-6 28851398 PMC5575848

[34] Mannan MA. On food and nutrition policy activities in the USA, Australia, and Norway. J Health Popul Nutr. 2004;22(2):191–202. 15473522

[35] Carey R, Caraher M, Lawrence M, Friel S. Opportunities and challenges in developing a whole-of-government national food and nutrition policy: lessons from Australia’s National Food Plan. Public Health Nutr. 2016;19(1):3–14. doi: 10.1017/S1368980015001834 26073889 PMC10271130

[36] de Alves KPS, Jaime PC. A Política Nacional de Alimentação e Nutrição e seu diálogo com a Política Nacional de Segurança Alimentar e Nutricional. Cien Saude Colet. 2014;19(11):4331–40. doi: 10.1590/1413-812320141911.08072014 25351300

[37] Pierre J, Peters BG. Governance, politics and the state. 2nd ed. Red Globe Press; 2020. pp. 95–201.

[38] Fanzo J, Davis C. Global Food Systems, Diets, and Nutrition: Linking Science, Economics, and Policy. Cham: Palgrave Macmillan; 2021. doi: 10.1007/978-3-030-72763-5

[39] World Bank. Breaking Silos: Cross-Sector Collaboration in Public Policy. Washington, D.C.: World Bank; 2023. https://www.worldbank.org

[40] Gomes M, Pereira MSS. Participação social e políticas públicas no Brasil: desafios e perspectivas. Rev Bras Cienc Polit. 2023;42.

[41] Mariath AB, Martins APB. Atuação da indústria de produtos ultraprocessados como um grupo de interesse. Rev Saude Publica. 2020;54:107. doi: 10.11606/s1518-8787.2020054002127 33146298 PMC7593022

[42] ACT Promoção da Saúde, Instituto Brasileiro de Defesa do Consumidor. Dossiê Big Food: Como a indústria interfere em políticas de alimentação. São Paulo. 2022. https://actbr.org.br/uploads/arquivos/DOSSIE-BIG-FOOD_Como-a-industria-interfere-em-politicas-de-alimentacao_ACT_IDEC_2022.pdf

[43] Albiero M, Amaral L. Dossiê Big Food 2.0: Como a indústria interfere em políticas de alimentação. São Paulo: ACT Promoção da Saúde. 2024. https://naoengulaessa.org.br/wp-content/uploads/dossie-big-food-v2.pdf

[44] Iverson SC, Iverson KL, Kevany JP. Food and nutrition policy formulation: a Delphi approach to establishing basic principles. Food Policy. 1979;:26–34.

[45] Latu C, Moodie M, Coriakula J, Waqa G, Snowdon W, Bell C. Barriers and facilitators to food policy development in Fiji. Food Nutr Bull. 2018;39(4):621–31. doi: 10.1177/0379572118797083 30486677

[46] Ulucanlar S, Lauber K, Fabbri A, Hawkins B, Mialon M, Hancock L, et al. Corporate political activity: taxonomies and model of corporate influence on public policy. Int J Health Policy Manag. 2023;12:7292. doi: 10.34172/ijhpm.2023.7292 37579378 PMC10462073

[47] OPAS. Prevenção e gestão de conflitos de interesse em programas de nutrição no âmbito nacional. 2022. https://iris.paho.org/handle/10665.2/55947

[48] Yeatman HR. Food and nutrition policy at the local level: Key factors that influence the policy development process. Critical Public Health. 2003;13(2):125–38. doi: 10.1080/0958159031000097625

[49] Fanzo JC, Graziose MM, Kraemer K, Gillespie S, Johnston JL, de Pee S, et al. Educating and training a workforce for nutrition in a post-2015 world. Adv Nutr. 2015;6(6):639–47. doi: 10.3945/an.115.010041 26567189 PMC4642431

[50] Villela DAM, Gomes MFDC. O impacto da disponibilidade de dados e informação oportuna para a vigilância epidemiológica. Cad Saude Publica. 2022;38(7):e00115122. doi: 10.1590/0102-311XPT115122 35894367

