# Supplementary material for: Facilitators and barriers to the formulation of public policies on food and nutrition: A scoping review
Source: PLOS Glob Public Health. 2026 Feb 23;6(2):e0005972. doi: 10.1371/journal.pgph.0005972 (PMC12928400; doi:10.1371/journal.pgph.0005972)
Supplement: S1 Text — (DOCX) [file pgph.0005972.s001.docx]

**S1 Text.** Search strategies used in online databases to identify eligible studies.

| **Database** | **Strategies** |
| --- | --- |
| Medline - PUBMED | (("nutrition policy"[MeSH Terms]) OR ("nutrition policy"[Title/Abstract:~4] OR "food policy"[Title/Abstract:~4] OR "diet policy"[Title/Abstract:~4] OR "nutrition action"[Title/Abstract:~4] OR "food action"[Title/Abstract:~4] OR "diet action"[Title/Abstract:~4] OR "nutrition plan"[Title/Abstract:~4] OR "food plan"[Title/Abstract:~4] OR "diet plan"[Title/Abstract:~4])) AND (("policy making"[MeSH Terms]) OR (“health plan implementation"[MeSH Terms]) OR (“policy making”[Title/Abstract] OR “policy evaluat*”[Title/Abstract] OR “policy develop*”[Title/Abstract] OR “policy statement*”[Title/Abstract] OR “policy strateg*”[Title/Abstract] OR “plan implement*”[Title/Abstract] OR “policy plan*”[Title/Abstract] OR “action plan*”[Title/Abstract])) |
| SCOPUS | ( ( TITLE-ABS-KEY ( ( nutrition* OR food OR diet* ) W/4 ( polic* OR action OR plan* ) ) ) ) AND ( ( TITLE-ABS-KEY ( "policy making" OR "policy evaluat*" OR "policy develop*" OR "policy statement*" OR "policy strateg*" OR "plan implement*" OR "policy plan*" OR "action plan*" ) ) ) AND NOT INDEX ( medline ) |
| WEB OF SCIENCE | #1 TS=((nutrition* OR food OR diet*) NEAR/4 (polic* OR action OR plan*))  #2 TS=(“policy making” OR “policy evaluat*” OR “policy develop*” OR “policy statement*” OR “policy strateg*” OR “plan implement*” OR “policy plan*” OR “action plan*”)  #3 #2 AND #1 |
| EMBASE | #1 'nutrition policy'/exp  #2 ((nutrition* OR food OR diet*) NEAR/4 (polic* OR action OR plan*)):ti,ab  #3 #1 OR #2  #4 'policy making'/exp/mj  #5 'health plan implementation'/exp/mj  #6 'policy making':ti,ab OR 'policy evaluat*':ti,ab OR 'policy develop*':ti,ab OR 'policy statement*':ti,ab OR 'policy strateg*':ti,ab OR 'plan implement*':ti,ab OR 'policy plan*':ti,ab OR 'action plan*':ti,ab  #7 #4 OR #5 OR #6  #8 #3 AND #6  #3 AND #6 AND [embase]/lim |
| LILACS | ((mh:("nutrition policy")) AND (mh:("policy making")) OR (mh:("health plan implementation")) OR ((policy making OR policy evaluat* OR policy develop* OR policy statement* OR policy strateg* OR plan implement* OR policy plan* OR action plan*))) |
| CINAHL | AB ( (nutrition* OR food OR diet*) w4 (polic* OR action OR plan*) ) AND AB ( (“policy making” OR “policy evaluat*” OR “policy develop*” OR “policy statement*” OR “policy strateg*” OR “plan implement*” OR “policy plan*” OR “action plan*”) ) |
